# Study of Elastin, Hydrolyzed Collagen and Collagen-like Products in a Tri-Layered Chitosan Membrane to Test Anti-Aging Skin Properties

**DOI:** 10.3390/ijms241311016

**Published:** 2023-07-03

**Authors:** Rocío Guerle-Cavero, Albert Balfagón-Costa

**Affiliations:** Pharmaceutical Chemistry Research Group, Institut Químic de Sarrià, Universitat Ramon Llull, 08017 Barcelona, Spain; rocioguerlec@iqs.url.edu

**Keywords:** chitosan, collagen, elastin, membrane, elasticity, moisture retention, pore

## Abstract

The use of animal testing in the cosmetic industry is already prohibited in more than 40 countries, including those of the EU. The pressure for it to be banned worldwide in the future is increasing, so the need for animal alternatives is of great interest today. In addition, using animals and humans in scientific research is ethically reprehensible. This study aimed to prove some of the anti-aging properties of elastin (EL), hydrolyzed collagen (HC), and two vegan collagen-like products (Veg Col) in a tri-layered chitosan membrane that was ionically crosslinked with sodium tripolyphosphate (TPP). In the first approach, as a way of representing different layers of a biological system, such as the epidermis and the two dermis sublayers, EL, HC, or Veg Col were independently introduced into the two inner layers (2L_(i+b)_). Their effects were compared with those of their introduction into three layers (3L). Different experiments were performed on the membrane to test its elasticity, hydration, moisture retention, and pore reduction at different concentrations of EL, HC, and Veg Col, and the results were normalized vs. a blank membrane. This new alternative to animal or human testing can be suitable for proving certain efficacy claims for active ingredients or products in the pharmaceutical, nutritional, and cosmetic fields.

## 1. Introduction

Animals have been widely used for biomedical research as an alternative to human beings, although it is important to exercise caution when extrapolating findings to human outcomes. Apart from these aspects, ethical concerns regarding the use of animals, together with the high costs, time-consuming protocols, lack of effective extrapolations, and lack of reproducibility in results, are important drawbacks in all basic research.

An EU Directive is taking specific actions with the goal of fully replacing the use of animals for scientific purposes [1].

In March 2013, the European Union started the first full ban on animal testing for cosmetics. For this reason, there is a need for the replacement of animal skin models, since they are forbidden in more than 40 countries for cosmetic purposes and are ethically regulated for medical purposes [2,3].

Some synthetic or natural biomaterial-based scaffold skin models that mimic the complex and stratified structure of human skin have been developed to replace the use of animals. Some skin models represent the epidermis, including EpiSkin^®^ (L’Oreal, Île-de-France, France), Epi-Derm^®^ (MatTek Corporation, Ashland, MA, USA), SkinEthic^®^ (SkinEthics, Lyon, France), epiCS^®^ (CellSystems, Troisdorf, Germany), Holoderm^®^, and Kaloderm^®^ (Seoul, Republic of Korea). Some other models represent the structure of the full thickness of human skin. Skin-on-a-chip models can also bring some other important aspects to light, such as the use of a vascular system, resulting in longer survival of the tissue [4]. However, these alternatives represent only some aspects of real human skin. Some other aspects, such as the skin barrier function, are poorly represented. Some major drawbacks of these models are that they can be time-consuming, dependent on expertise, and costly [5]. Hence, there is still much research with the aim of finding an alternative system in which the complexity of human skin can be achieved [6].

In our previous work [7], a tri-layered chitosan membrane that was ionically crosslinked with sodium tripolyphosphate (TPP) was created as a physical model in an attempt to simulate different skin layers with the aim of providing similar results to those obtained with human skin, but with strong simplifications. The use of living cells was avoided, as there was no goal of reproducing the conditions and complexity of human skin. The materials employed were cost-effective, and they could mimic several skin characteristics and properties, such as the different layers, skin pore size, elasticity, hydration, and moisture retention. In this work, two kinds of membranes were tested: a base membrane and an activated membrane. In the second case, pores were mechanically created, as was already reported [7].

Ionic crosslinking was chosen, among other reasons, because this kind of crosslink is created between collagen fibers and proteoglycans in the extracellular matrix (ECM); thus, the components of our membranes had a similar structure [8]. These crosslinks confer structure to the skin. In contrast to ionic crosslinking, covalent crosslinking is formed in the skin between the amino acids that form proteins located in the major part of the ECM, which is mainly to give strength and elasticity to the skin.

Chitosan was chosen as the main scaffold biomaterial, and TPP was chosen as the crosslinker for many different reasons.

1. Chitosan is derived from chitin, and it is probably the second most abundant polymer in nature, behind only cellulose.
2. Chitosan has a similar structure to that of glycosaminoglycans (GAGs), an essential component of the ECM [9].
3. Chitosan is characterized by the presence of primary amino groups along its polymer backbone, which causes its structure to interact with different proteins, cells, and living organisms.
4. Chitosan can have different degrees of deacetylation (DDAs), from 50 to 100%, which determines its final properties [7].
5. Chitosan is obtained from sustainable chitin, as chitin is a byproduct of crustaceans in the food industry.
6. Chitosan is affordable compared to other biomaterials that can be naturally found in skin, such as collagen (Col), EL, and GAGs, such as hyaluronic acid.
7. TPP is non-toxic, simple to use, widely available, affordable, and an excellent ionic crosslinker of chitosan.
8. Chitosan is a well-known and well-studied biomaterial for artificial skin scaffolds [10].
9. This kind of membrane based on chitosan could have potential for use in biomedical applications. Thanks to its hemostatic, antimicrobial, and antifungal properties, chitosan could be a suitable option for burn and wound treatments [11,12].

In this research, EL and HC were selected with the aim of testing their efficacy in a Ch-based membrane [13,14]. EL and Col, together with GAGs, are some of the key components of the ECM. The ECM is a non-cellular, three-dimensional macromolecular network in which cells reside, and it is found in all tissues and organs [15,16].

EL and HC were introduced into the 3L of the membrane to test their efficacy in the entire membrane. The three layers of the membrane were intended to represent the epidermis and the two dermis sublayers, namely, the papillary dermis and the reticular dermis. For this reason, EL and HC were also independently introduced into the two inner layers, where they are naturally found in human skin [6].

EL, with a molecular weight of around ≈70 KDa, is the main component of elastic fibers. Its role is closely linked with that of Col; it provides stretching, recoil, and elasticity to the skin. It is also located in the dermal layer of the skin and makes up approximately 2–4% of the dry weight of the dermis in the skin of adults [17]. It is composed of alternating hydrophilic and hydrophobic segments [18,19,20].

In contrast to HC, EL, whether or not it is in its hydrolyzed form, has not been extensively employed in cosmetics or as a nutraceutical, despite some of its beneficial effects that have been found in skin, such as its improvement of skin elasticity in combination with Col and with some other biomaterials [21]. EL is rarely used in bioscaffolds, and it is mainly used for blending with other polymers, such as Col or GAGs [22,23,24]. It is mainly used as an additive for other scaffold materials, such as Col, due to its poor mechanical strength and availability. The MatriDerm^®^ matrix (Billerberck, Germany) is an example of this, where EL is used to modify Col to mimic elastic fibers in the native dermis [12].

The addition of EL to a Ch membrane can increase its elasticity and mechanical strength, improve hydrophilicity, and increase degradation rates [25]. Hydration of EL is necessary for obtaining elasticity. EL monomers are disordered and flexible in water. They can act as plasticizers in water by interacting with the water molecules that are bound to the main chain, favoring its movement. A hydrated elastin chain can form transient hydrogen-bonded turns. These turns are not random but, rather, dynamic conformation structures that confer flexibility and contribute to high disorder. Furthermore, the hydrophobic segments prevent the formation of large secondary structures that could block chain motion [26].

Col, with a molecular weight around ≈300 KDa, is produced by fibroblasts. It provides tensile strength to the skin [15]. It is located in the dermis, provides physical support to tissues, plays an important role in the structural and biological integrity of the ECM, and represents 75% of the dry weight of the skin. It is also an amphoteric macromolecule [27].

It was already reported that Ch was used in combination with Col due to its antibacterial properties and because it reduces the biodegradation of the scaffold [12]. Col can improve the tensile strength and elasticity of chitosan scaffolds depending on the dosage used [10,16,28].

Col, especially in its hydrolyzed form, is a well-known anti-aging ingredient that is used in cosmetics, pharmaceuticals, healthcare, and the beverage and food industries [29]. It is also a widely used biomaterial scaffold, and chemical crosslinking is the most suitable method for type I Col. However, most crosslinkers are expensive, difficult to manage, and cytotoxic, as with glutaraldehyde; in most cases, they cannot be applied alone, as in the case of N-hydroxysuccinimide (NHS) and 1-ethyl-3-(3-dimethylamino-propyl) (EDC) [30]. Col is used as a skin substitute in different commercial products, such as Integra^®^ (Princetown, NJ, USA), OrCel^®^ (Forticell Bioscience, New York, NY, USA), Promogran Prisma^®^ (3M, Two Harbors, MN, USA), and PuraPly^®^ (Organogenesis, Inc., Canton, MA, USA), to name a few [12].

By combining Col with Ch, two kinds of interactions take place—hydrogen bonding and electrostatic interactions—and these reinforce the mechanical strength [31]. According to Taravel et al. [20,32], there is a weak interaction between Ch and Col that forms polyanion-polycation complexes (–NH_3_^+^ from Ch with the –COO^−^ group from Col) in slightly acidic solutions, although this is obstructed by Col gel formation.

In Col, different hydrogen bonds can be formed, such as those between chains by hydroxyproline –OH groups, those between other side groups, those involved in the formation of fibrils, and those with the –OH and NH_2_ groups from Ch. Additionally, hydrogen bonds can be formed between the end groups of –COOH and NH_2_ of Col and the –OH and –NH_2_ groups of Ch, as Ch contains large numbers of –OH groups [33].

With an excess of Ch, a hydrogen-bonding-type complex can be formed in addition to an electrostatic complex, whereas a self-crosslinking phenomenon is induced with an excess of Col [16,34]. However, the ionic interaction of TPP with Col chains is small since the number of exposed amino groups in Col chains is very small.

In this research, different tests were performed to test the anti-aging efficacy of EL, HC, and two Veg Col products.

- Elasticity is the ability of the skin to return to its resting state after displacement [35].
- Hydration is a complex process composed of two mechanisms—the barrier effect or moisture retention—and is the ability of a system to avoid water loss. In the skin, this is known as transepidermal water loss (TEWL). On the other hand, humidity absorption is the capacity to absorb water from the environment [36,37,38].
- The pore reduction effect is the capacity of a material to reduce its pore size compared to that in its initial state.

From the results of this study, it can be stated that these membranes are sensitive to specific properties.

1. These properties are sensitive to the concentrations of the active ingredients and Veg Col products.
2. It was demonstrated that the effects of some active ingredients, namely EL, HC, and two Veg Col products, were evidenced in terms of pore reduction, water permeation, elasticity tests, swelling, and moisture retention. Hence, anti-aging claims could be proven.
3. The effects of the active ingredients and Veg Col products on the previous properties varied depending on the layer in which they were introduced.

## 2. Results and Discussion

### 2.1. Pore Quantification

Pores have different sizes depending on age, sex, ethnicity, and body area. Facial skin pores are a great concern for the beauty market, and their size may vary for different reasons, such as high sebum excretion, decreased elasticity around pores, an increase in hair follicle volume, and dehydration [39,40,41].

Table 1 shows the pore areas that were measured for activated membranes with extreme concentrations in order to see their differences with respect to blank activated membranes. It can be seen the different mean pore areas for some EL and HC studied concentrations and the percentage of pore reduction versus blank containing the active ingredient in 3L or M2L_(i+b)_ according to Equation (4). All concentrations appearing in this work are expressed as *w*/*w*. Other expressions of concentrations will be specifically detailed.

As previously reported by Flament et al. [39], facial pore size varies depending on ethnicity, from 15,000 μm^2^ for the Chinese ethnicity to 92,500 μm^2^ for the Brazilian ethnicity. In the case of Asiatic facial pores, the pores are the smallest. For Caucasians, the skin values are in the middle, ranging between 40,000 and 55,000 μm^2^ (calculated as the area of a circle) [42]. All of these pore sizes can be studied with these kinds of membranes; the mean pore size was 39,045 µm^2^ for the blank activated membrane, which had even lower values than those of Caucasian skin at some concentrations of EL and HC.

When EL was included in the activated membrane, the effect on pore size reduction was most evident at the highest concentration, 0.28%, with the highest effect in both 3L and 2L_(i+b)_; there was a pore reduction of −18%. A possible explanation for this having the same effect in both kinds of membranes could be that EL was able to diffuse from the inner to the top layer in 2L_(i+b)_, thus producing the same effect as that in 3L. When the EL content was low (0.085%), the effect was a bit more pronounced when EL was included in 3L (−4.9%), but its effect at this low concentration was hardly perceived when it was included in 2L (−0.48%). In any case, the pore size reduction was directly proportional for both 3L and 2L_(i+b)_.

In the case of HC, the effect on pore size reduction was also evidenced the most with the highest concentration (10%); again, the greatest effect on pore reduction was in 3L (−22%), which was in contrast to when it was included in 2L_(i+b)_ (−8.5%). The pore size reduction was also directly proportional to the concentration for both 3L and 2L_(i+b)_.

Nowadays, noticeable pore size is a great concern for consumers [43]. Hence, these results can be helpful for claiming the beneficial effects of these active ingredients or products on reducing pore size. The effect of HC on pore size reduction was also evidenced in previous scientific reports [44].

**Figure 1:**
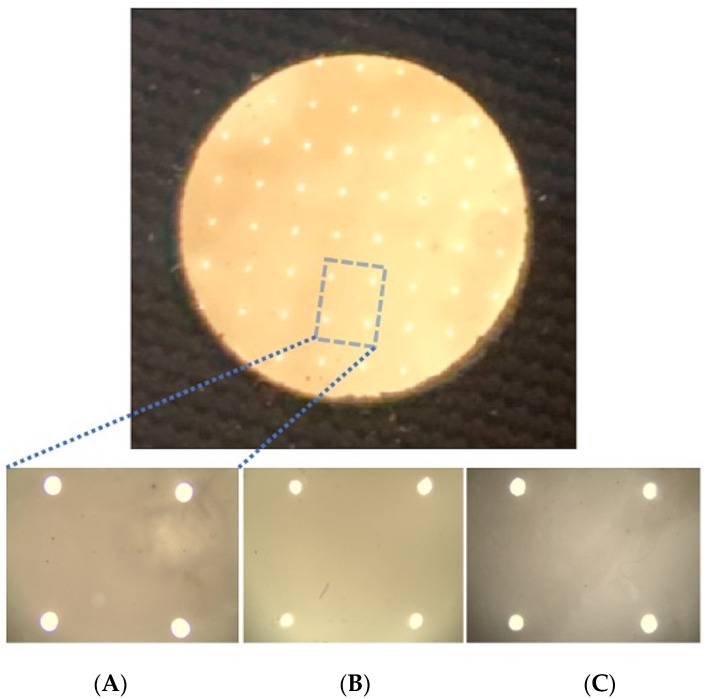
Pore visualization with an optical microscope in the activated membranes with a semi-plan 4 × 0.10 oil-immersion objective with a wide field of 0.45: (**A**) blank; (**B**) with EL included at 0.28% in 3L; (**C**) with HC included at 10% in 3L.

In Figure 1, there is visual evidence of the most extreme cases of pore reduction for both the EL- and HC-activated membranes in comparison with the activated blank.

When looking at the images obtained with the microscope, the pore size reduction was visually evident, and it can also be stated that the shapes of the pores varied arbitrarily.

A possible reason for these two phenomena could be the kinds of interactions between Ch, HC, or EL and TPP. When layers were composed of only Ch, well-shaped and firm channels were formed. Nevertheless, when HC or EL were included, some interactions could occur, as they could be located between adjacent molecules of Ch and TPP to produce arbitrary links. As a consequence, different effects could be promoted, such as increasing plasticity or disrupting the Ch structure. In addition, an increase in swelling could be achieved because of the nature of Col and EL, as the membranes were in contact with water during their formation. Consequently, water molecules could diffuse into the membrane and into the pores’ surroundings. This fact could promote the disruption of the intermolecular forces that tightly held the polymer chains. Therefore, as a possible consequence of these two phenomena, the formation of irregularities in the channels and reductions in pore size could take place.

### 2.2. Permeation Tests

As a means of studying the permeability, 30 g of water was permeated through an activated membrane in a Franz diffusion cell. During the permeation tests, the blank activated membranes reached the highest permeation of water in the first minute, and the 30 g of water was completely permeated in 2.9 min on average. For this reason, the quantity of water permeated within the first minute was used to compare the pore sizes. The reference value for the experimental results was the mean water permeation through the blank activated membranes for one minute (15 g). The mean water permeation (%) of the different experiments was calculated according to Equation (5).

#### 2.2.1. Permeability of the EL-Activated Membranes

Three different concentrations of EL were studied for the water permeation tests. The lowest one, 0.085%, fell within the margins of the EL content in dermal skin.

**Figure 2:**
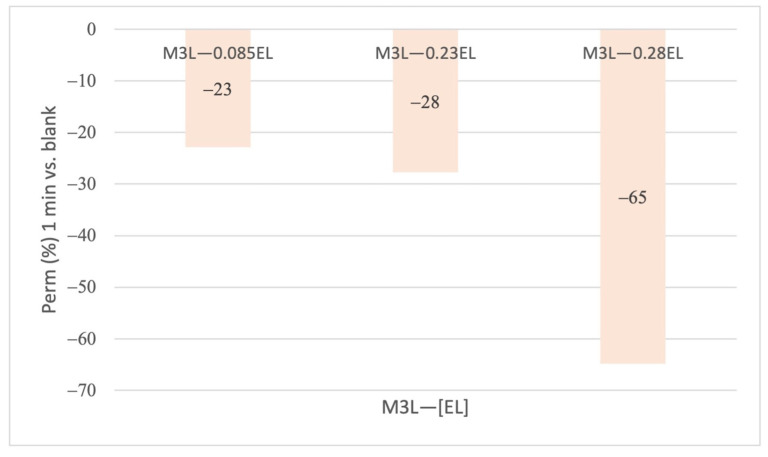
Mean reduction (%) in water permeation at 1 min for the activated membranes containing EL at 0.085, 0.23, and 0.28% in 3L vs. the blank activated membrane.

**Figure 3:**
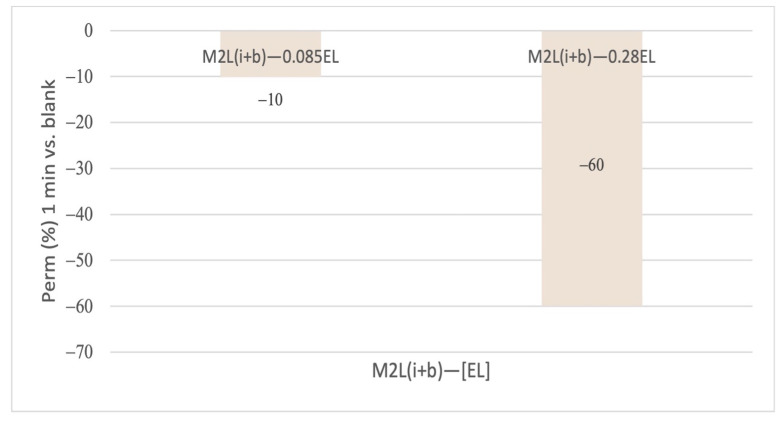
Mean reduction (%) in water permeation at 1 min for the activated membranes containing EL at 0.085 and 0.28% in 2L_(i+b)_ vs. the blank activated membrane.

In Figure 2 and Figure 3, the differences that were observed when EL was included in 3L and 2L_(i+b)_, respectively, can be seen.

As shown in Figure 2, a 65% reduction in permeation was observed with 0.283% EL, which was a reduction in permeation by more than half with respect to the blank.

As shown in Figure 3, when EL was included in 2L_(i+b)_, a 60% water permeation reduction was observed at the highest concentration of EL (0.283%), showing the high power of EL in water permeation reduction, despite only being included in 2L_(i+b)_.

These results show that EL had a very strong effect on the reduction in water permeation. It worked in a concentration-dependent manner for both kinds of membranes (3L and 2L_(i+b)_). The effect of water permeation reduction was slightly clearer when EL was introduced in 3L than in 2L_(i+b)_, as there was no EL in the first layer. Thus, the water permeation reduction was less evident.

#### 2.2.2. Mean Permeability of the HC- and Veg-Col-Activated Membranes

In the case of HC, eight different concentrations were studied to see their effects on water permeation. In addition, two different Veg Col products were also studied at the concentration recommended by the supplier (2%).

**Figure 4:**
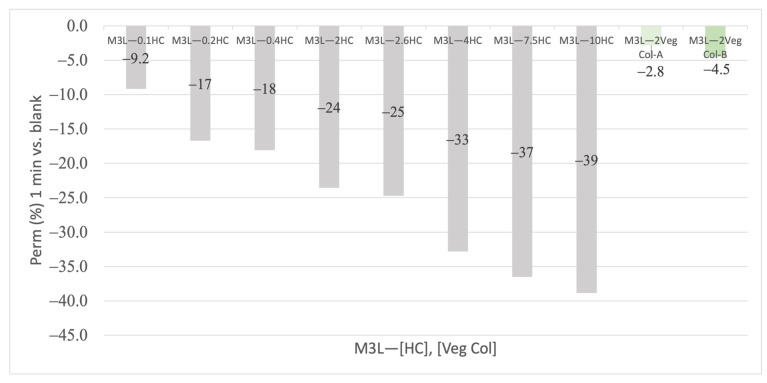
Mean reduction (%) in water permeation at 1 min for the activated membranes containing HC at 0.1, 0.2, 0.4, 2, 2.6, 4, 7.5, and 10% represented in dark grey or, Veg Col type A and B represented in different green intensities at 2.0%, in 3L vs. the blank activated membrane.

**Figure 5:**
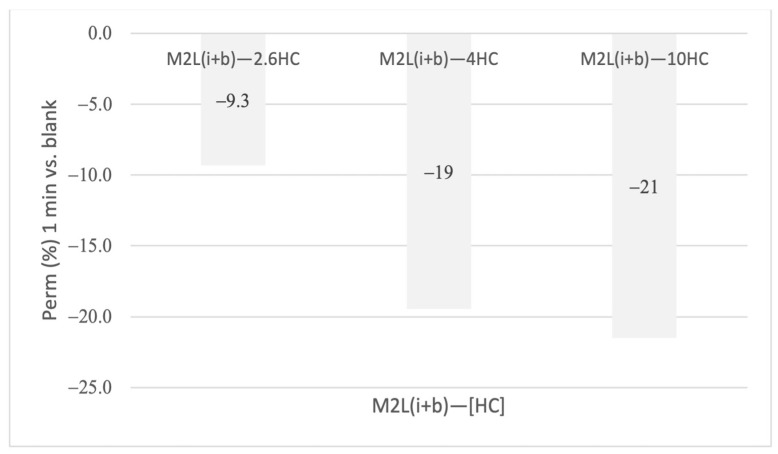
Mean reduction (%) in water permeation at 1 min for the activated membranes containing HC at 2.6, 4, and 10% in 2L_(i+b)_ vs. the blank activated membrane.

In Figure 4 and Figure 5, the differences observed when HC was just studied in 3L or 2L_(i+b)_ at some concentrations can be seen.

As shown in Figure 5, the reduction in water permeation was, again, not as strong as when HC was introduced in 3L, but the effect was also concentration dependent.

Well-arranged values were observed for the eight different concentrations that were studied, as the water permeation decreased from −9.2% to −39%. It should be noted that HC is a small molecule compared to EL. Hence, although the concentration of HC was higher than that of EL, it was foreseeable that the water permeation reduction would not have as much of an impact as that of EL (from −23% to −65%), as the latter has longer hydrophobic segments.

In the case of Veg Col, the effect of water permeation reduction was barely noticeable in comparison with the HC of animal origin.

### 2.3. Relationship between the Mean Water Permeability and the Mean Pore Reduction

In Table 2, the relationship between the mean percentage of water permeation reduction and the mean percentage of pore reduction is shown for both active ingredients (EL and HC) that were studied.

When comparing both active ingredients, it can be seen that in the case of EL, the water permeation reduction was greater than that with HC for similar values of pore reduction. The difference increased at high concentrations of both active ingredients. An explanation for this divergence could be because of the nature of EL. EL contains more hydrophobic segments as it has a longer chain compared to that of Col, which is hydrolyzed. This issue may impact the water permeability by slowing down the permeation process, despite the fact that the effective pore size obtained with EL was not much smaller than that obtained with HC.

From the results obtained, it can be seen that the greatest pore reduction was achieved when 10% HC was added to 3L, and the greatest water permeation reduction was obtained when 0.28% EL was added to 3L.

### 2.4. Rheological Tests

All elastic modulus results are mean values of G′ (Pa) that were obtained within 2 min. For blank rheological tests, it was evident that the elasticity results were very sensitive to the room temperature during membrane formation. All membranes studied for the rheological tests were base membranes.

**Figure 6:**
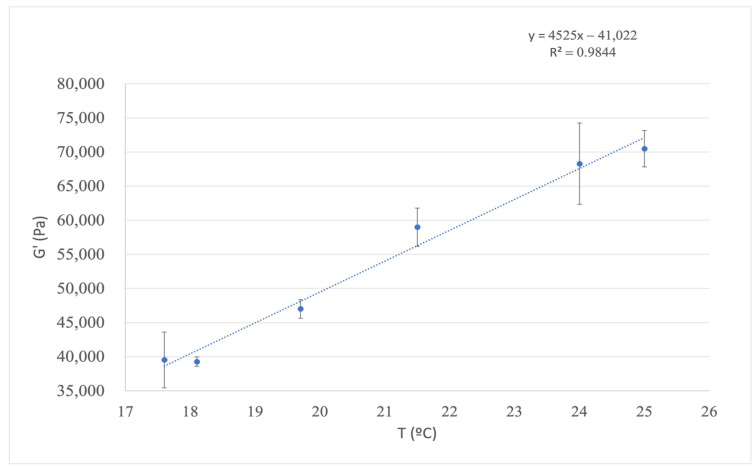
Mean elastic moduli of the blank membranes at different temperatures.

As can be observed in Figure 6, the elasticity of the blank membranes followed a good model of linear regression (R^2^ = 0.9844) with respect to temperature in the range of room temperatures studied (from 17.5 to 25 °C).

**Figure 7:**
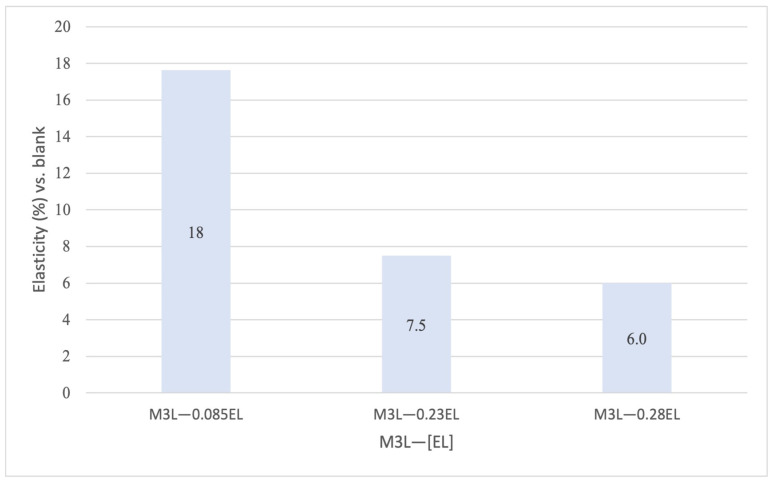
Mean elasticity (%) for EL membranes normalized vs. blank membranes with EL included at 0.085, 0.23, and 0.28% in 3L.

**Figure 8:**
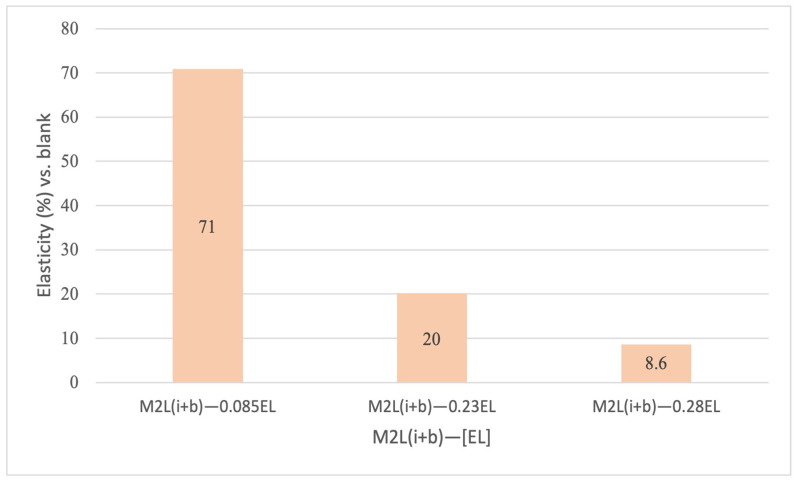
Mean elasticity (%) for EL membranes normalized vs. blank membranes with EL at 0.085, 0.23, and 0.28% included in 2L_(i+b)_.

**Figure 9:**
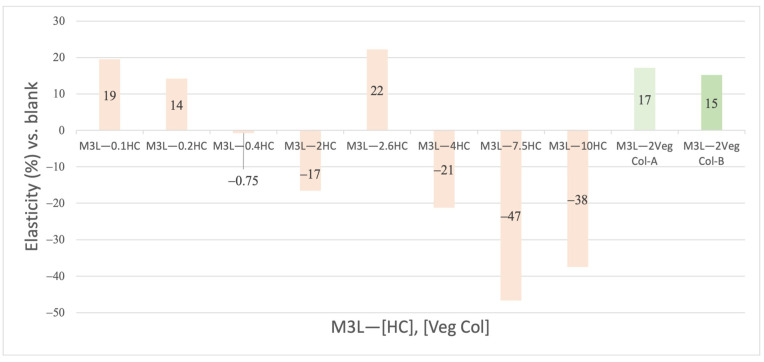
Mean elasticity results (%) for HC and Veg Col type A and B membranes normalized vs. blank membranes with HC at 0.1, 0.2, 0.4, 2, 2.6, 4, 7.5, and 10%, represented in orange, and with Veg Col type A and B at 2% represented in different green intensities, in 3L.

**Figure 10:**
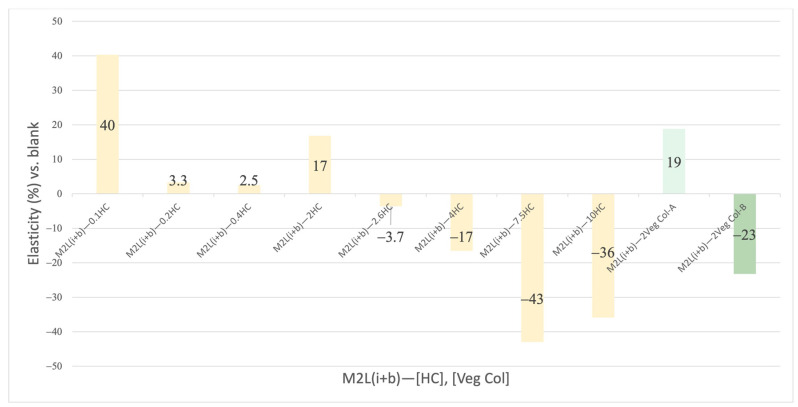
Mean elasticity results (%) for HC and Veg Col type A and B membranes normalized vs. blank membranes with HC at 0.1, 0.2, 0.4, 2, 2.6, 4, 7.5, and 10%, represented in yellow, or with Veg Col type A and B at 2%, represented in two green intensities, in 2L_(i+b)_.

All elastic modulus results, which are shown in %, of the membranes containing the different active ingredients or products were normalized (Equation (7)) with respect to their blank membrane interpolation in the linear regression model (Equation (6)), as shown in Figure 7, Figure 8, Figure 9 and Figure 10.

In Figure 7, it can be observed that, when EL was added to 3L, the elasticity decreased as the EL concentration increased.

In Figure 8, it is shown that, when studying these three concentrations of EL in 2L_(i+b)_, the tendency was the same, causing the percentage of elasticity to decrease as the EL concentration increased.

However, when comparing the same concentration of EL in 3L and 2L_(i+b)_, the effect on elasticity was increased in 2L_(i+b)_; the greatest difference was at the lowest concentration in the solution (0.085%), and this was from 18% (3L) to 71% (2L_(i+b)_). This concentration (0.085% EL) was equal to 2.6% EL in dry weight. This concentration fell within the margins of the content of EL in human dermal skin (2–4% in dry weight). This fact suggests that the EL content in human skin has the optimal concentration for achieving the highest elasticity capacity. For the last concentration that was studied (0.28%), there was almost no difference between the two kinds of membranes (3L or 2L_(i+b)_).

From the results obtained for EL, it can be seen that for the three concentrations studied, EL always produced an increase in elasticity, although it was inversely proportional to the concentration. An elastic booster effect was obtained when EL was included in 2L_(i+b)_ at all concentrations studied.

A possible explanation for the increase in elasticity could be that when EL is in contact with Ch, a unique hydrogen binding site between EL and Ch is achieved. Thus, it could break down the inter- and intramolecular hydrogen bonding networks of Ch. So, hydrophobic segments of EL that can form disordered assemblies’ structures may hinder the formation of intermolecular hydrogen bonding networks. Thus, this would favor the motion of Ch chains. This effect reached its maximum at the lowest concentration of EL, 0.085%. When the EL content was increased, what could have happened is that fewer hydrogen bonding sites could be achieved between Ch and EL, as there could be saturation of EL, so a decrease in elasticity was observed.

As shown in Figure 9, at 0.1% and 0.2% HC, according to literature, when there is an excess of Ch, a hydrogen-bonding-type complex and an electrostatic complex could form between Ch and HC, thus producing some elasticity in the membrane. Apart from these two kinds of complexes, a small interaction between TPP and HC could be produced, which may have also helped to increase the elasticity.

For higher concentrations, we have considered the following hypothesis. When the concentration of HC began to increase from 0.4% to 2%, a more disorganized network was produced, resulting in a reduction in elasticity. When the concentration of Ch was almost equal to that of HC, it seemed that a more ordered network was formed, and this was able to create more bonds between Ch, TPP, and HC. Hence, an increase in elasticity was observed. At higher concentrations, from 4 to 10% HC, a more disorganized network was achieved, as the excess of HC disturbed the links between Ch and TPP. Hence, a disruption in molecules’ alignment was caused, leading to a decrease in elasticity. At 7.5% HC, the greatest effect on elasticity reduction (−47%) was obtained.

An inversely proportional relationship between Ch and HC concentrations was also observed when the peak of elasticity found when both concentrations were almost equal was excluded.

For both kinds of Veg Col that were studied, a noticeable effect on elasticity was found at the concentration of 2% recommended by the supplier. In the case of Veg Col-A, the “collagen-like” active ingredient was combined with glycerin. Glycerin is known to have good plastic properties in the skin [45]. When it is combined with Ch, its single hydrogen bonding site can break down the inter- and intramolecular hydrogen bonding networks of, thus leaving hydrophobic C-H ending groups to limit intermolecular hydrogen bond formation and allowing free motion of the chitosan chains [46]. According to the supplier of Veg Col-A, glycerin was found in the product at 1.4% when using 2% Veg Col-A. Some elasticity tests were performed by adding 1.4% glycerin to the blank solution in order to check its influence on elasticity. There was a 16% increase in elasticity with respect to the blank. Hence, it seems that almost all increases in elasticity with Veg Col-A (17%) could be attributed to the plastifying effect of glycerin. In the case of Veg Col-B, the elasticity value of 15% could only be attributed to the active ingredient “vegan Col”.

As shown in Figure 10, an even greater increase in elasticity was observed at 0.1% HC (40%) in 2L_(i+b)_ than with the same concentration in 3L (19%). Both hydrogen bonding and electrostatic complexes could also be formed here. Our hypothesis in this case was that, apparently, when HC was included in 2L_(i+b)_ at the lowest concentration (0.1%), it did not disturb the interaction of Ch and TPP. At the same time, HC could also form links with Ch from the same layer and from the top layer. A possible explanation could be that, in this case, the molecules could be organized in a way that favored linkages, thus acting as a dominant force for some other kinds of links in opposite directions, which could disturb the interaction. It seems that the HC located in the intermediate layer may still have had carboxylic groups that were able to react with free amino groups and –OH from the Ch stock solution in the last layer by forming more hydrogen bonds. It seems that in 3L at 0.1% HC, the molecules were a bit more disorganized than those in 2L_(i+b)_, which had lower elasticity values (19%).

However, when the concentration of HC was 0.2 or 0.4%, the effect on elasticity was reduced to almost negligible. At 2% HC, a peak in elasticity was observed. A possible explanation of this effect could be that the diffusion of HC to the top layer may have had a similar effect on elasticity to that seen for the concentrations of 0.1 or 0.2% HC in 3L, suggesting that a similar concentration could diffuse to the top layer. Hence, the elasticity had a similar value to that obtained with those two concentrations (17%). For cases in which there was an excess of HC, the same effect on 3L was also visualized in 2L_(i+b)_, but with slightly lower values of elasticity, as the concentration of HC in the whole membrane was not as high as in 3L. In the case of Veg Col-A, the elasticity was found to be similar for both kinds of membranes, and the positive effect of elasticity due to glycerin was observed in both cases (17 and 19%, respectively). For Veg Col-B, based only on the effect of the “collagen-like” active ingredient, the elasticity decreased when it was included in the two inner layers, and it was even lower than in the blank membrane (−23% in 2L_(i+b)_ in contrast to 15% in 3L).

In the 2L_(i+b)_ membranes, as shown in Figure 10, an inversely proportional relationship between the Ch and HC concentrations was found, except for the peak of elasticity that was found when Ch was at 2.5% and HC was at 2%.

These elasticity results clearly demonstrate that EL, HC, and the two Veg Col products had an influence on the elasticity of the membrane, as they increased or decreased its value depending on the concentration used. It was also evidenced that when the active ingredient or Veg Col product was included in 2L_(i+b)_, the elasticity values for the lowest concentration were boosted in comparison with the membrane in which the active ingredient or Veg Col product was included in 3L.

Some similarities with our results were found in the elasticity results obtained by Hydalgo-Vicelis et al. [28], where membranes were prepared in the form of films with the solvent evaporation technique and were composed of Ch and Col, which were crosslinked by a covalent crosslinker (EDC). In their case, different proportions of both Ch and Col were combined, unlike in our study, where the concentration of Ch was almost constant, but only the HC concentration varied. When a low concentration of Col was used, the elasticity obtained had the highest value, but, as the Col concentration increased, the elasticity values decreased. In this case, although they did not keep the Ch concentration constant, a tendency similar to that in our study was found despite the use of different types of crosslinkers.

According to Martínez et al. [16], they also tested EDC and TPP as crosslinkers for Ch membranes. In their case, the membranes were prepared by freeze-drying and combining three different proportions of Ch and Col. For TPP-crosslinked membranes with Col in the minority, the highest elasticity value was obtained. When the concentration of Col was equal to that of Ch, they found a reduction in the elasticity value, which was not in concordance with the values that we obtained. When the quantity of Col was in the majority, the elasticity increased, but not as much as when it was in the minority. For EDC-crosslinked membranes and when using a combination of both TTP and EDC, they showed a directly proportional concentration-dependent behavior as the Col concentration increased with the three concentrations that were studied.

These results show that, despite the use of the same crosslinker, depending on the technique of preparation of the membrane and the combination of concentrations of both Ch and Col, the tendencies of the elasticity results can substantially vary.

### 2.5. Swelling Index

**Figure 11:**
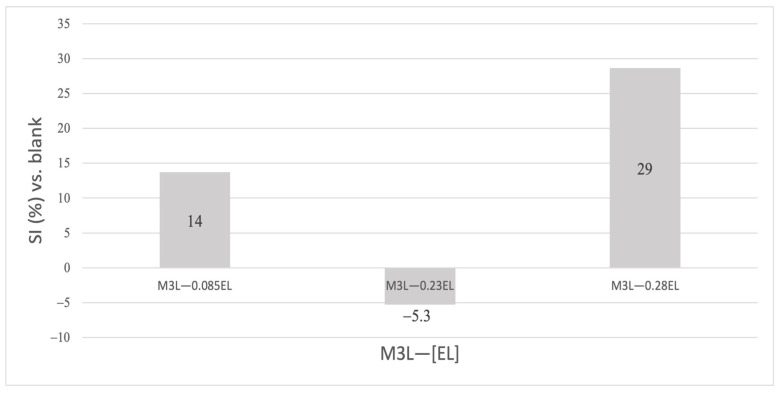
Mean swelling indexes (%) for EL membranes normalized vs. blank membranes with EL at 0.085, 0.23, and 0.28% in 3L.

**Figure 12:**
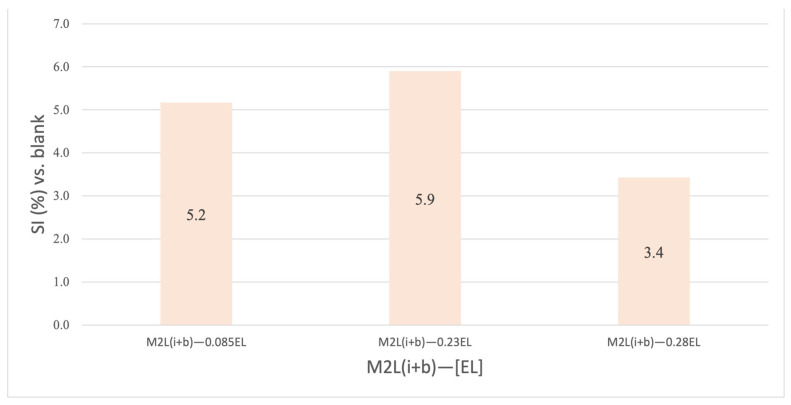
Mean swelling indexes (%) for EL membranes normalized vs. blank membranes with EL at 0.085, 0.23, and 0.28% in 2L_(i+b)_.

**Figure 13:**
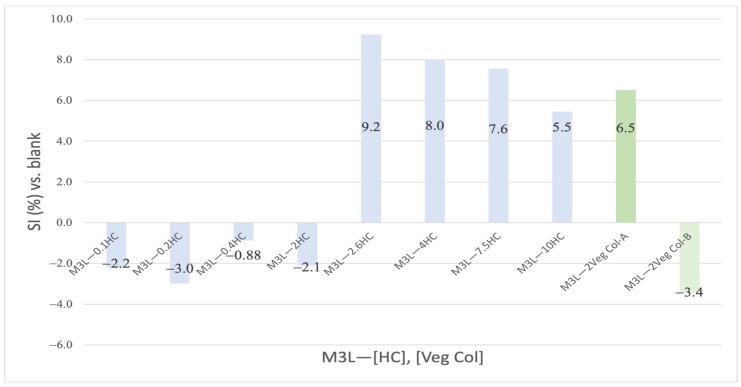
Mean swelling indexes (%) of HC and Veg Col type A and B membranes normalized vs. blank membranes with HC at 0.1, 0.2, 0.4, 2, 2.6, 4, 7.5, and 10% represented in blue, and Veg Col type A and B represented in different green intensities at 2% in 3L.

**Figure 14:**
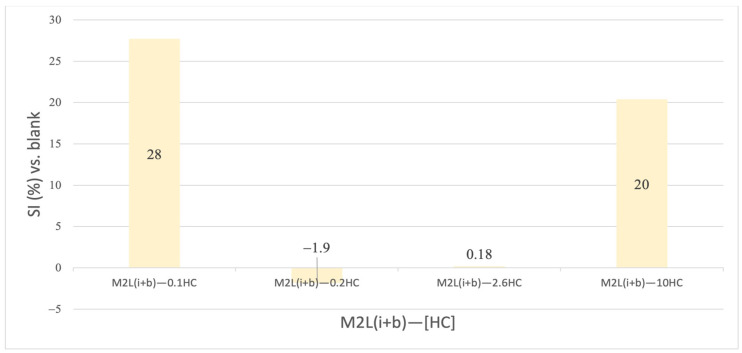
Mean swelling indexes (%) of HC normalized vs. blank membrane with HC at 0.1, 0.2, 2.6, and 10% in 2L_(i+b)_.

The swelling index (SI) of the different kinds of tri-layered crosslinked base membranes was calculated according to Equation (8) for different lengths of PBS baths, and the dry state of each membrane was taken as a reference. The SI obtained for the blank membranes was 114%. All SIs of the membranes containing different active ingredients or Veg Col products studied were normalized (Equation (9)) with respect to the blank membrane, as shown in Figure 11, Figure 12, Figure 13 and Figure 14.

In Figure 11, it can be seen that the swelling effect of EL was evidenced at 0.085%, and at 0.283% EL, the highest swelling effect was obtained (29%) in 3L. The intermediate concentration of 0.23% EL in 3L did not have any positive effect on swelling (−5.3%).

As shown in Figure 12, when EL was only included in 2L_(i+b)_, the swelling effect at extreme concentrations was reduced compared to that in 3L, but it was higher at the intermediate concentration compared to that in 3L (5.9%). For the three concentrations of EL included in 2L_(i+b)_, similar SIs were observed.

As shown in Figure 13, swelling effects were not observed at low concentrations—from 0.1 to 2% HC—when this ingredient was introduced into 3L. However, a certain SI was obtained at concentrations at which there was an almost equal or greater concentration of HC with respect to Ch—from 2.6 to 10% HC. It should also be clearly stated that very similar values were obtained in that range, and they were slightly higher when the concentrations of Ch and HC were almost equal (SI of 9.2%). There was also a tiny tendency toward swelling reduction from 2.6 to 10% HC.

A positive SI was obtained for Veg Col-A but not for Veg Col-B. It should be considered that 1.4% glycerin was contained in the 2% Veg Col-A used. Glycerin is an ingredient that is commonly used in topical products due to its good properties as a humectant [47]. A swelling test—not shown in Figure 14—was performed for a membrane containing 1.4% glycerin in 3L to determine the effect of glycerin. An SI of 7.0% with respect to the blank was obtained. Thus, it seems that the SI of Veg Col-A was only achieved because of glycerin’s effect.

As shown in Figure 14, a noticeable effect on swelling was achieved for extreme concentrations (0.1 and 10% HC: 28 and 20%, respectively). No effects were observed for the two intermediate concentrations studied (0.2 and 2.6% HC). It can be highlighted that the effect of 10% HC was much higher in 2L_(i+b)_ (20%) than in 3L (5.5%).

As has already been stated in the scientific literature, the swelling behavior of a Ch membrane crosslinked with TPP and with the inclusion of EL or Col could be influenced by the following:

1. The degree of crosslinking; increasing the crosslinking density, which produces an increase in elasticity, could lead to a decrease in swelling due to the formation of a more compact structure [48,49].
2. Increasing the concentrations of both EL and Col could also lead to an increase in swelling, as they contain some hydrophilic regions that can interact with water molecules, thus promoting swelling [16,50,51]. Another aspect could be that both active ingredients could produce a more porous structure in the membrane and a more organized and structured network that could favor water absorption [52].

### 2.6. Moisture Retention

Moisture retention (MR) was measured in the base membranes containing the active ingredient or Veg Col products in 3L due to the conditions of this test. The water loss (in %) for the blank membranes was 60% at 15 min and 80% at 30 min. The moisture retention percentage for each membrane under study, normalized versus blank membrane, was calculated according to Equation (10).

**Figure 15:**
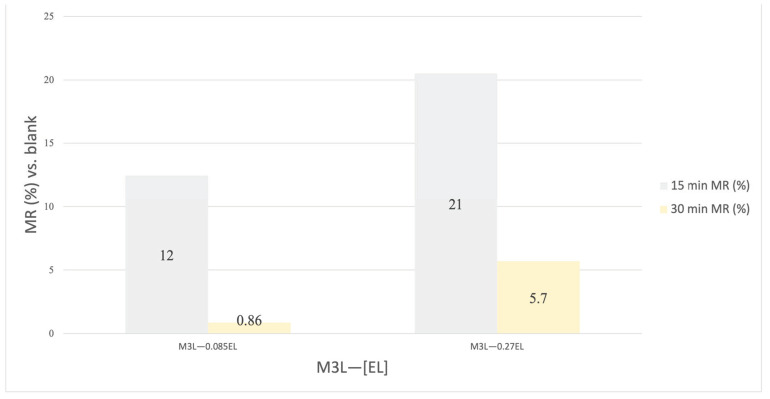
Mean moisture retention (%) at 15 and 30 min of the EL vs. blank membrane with EL at 0.085 and 0.27% in 3L.

As shown in Figure 15, strong moisture retention abilities were observed for both concentrations of EL at 15 min; the value was higher (21%) at the highest concentration (0.27%). A lower moisture retention was observed at 30 min than at 15 min. These results suggest that EL has good short-term water retention abilities but a poor ability to retain moisture in the long term.

**Figure 16:**
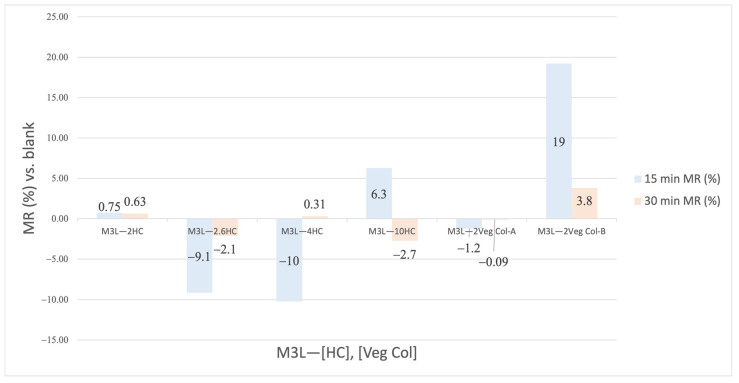
Mean moisture retention (%) of HC at 2, 2.6, 4, and 10% and both kinds of Veg Col type A and B at 2% vs. the blank membrane at 15 and 30 min, in 3L.

As shown in Figure 16, there was a negligible or negative effect on moisture retention for the studied concentrations of 2, 2.6, and 4% HC. This could mean that HC was able to disrupt the Ch structure, thus favoring water release.

However, some moisture retention was observed for 10% HC at 15 min (6.3%). This could mean that this kind of membrane was more compact and closed than that in which Ch was in the majority. Water could diffuse easily because a more porous structure could be formed at 10% HC, and it was kept inside, so water retention was favored.

For the two types of vegan Col studied, Veg Col-B presented a strong moisture retention power in the short term (19%), unlike Veg Col-A, which had no positive effects (−1.2%). The moisture retention power of Veg Col-B (19%) was similar to that of the highest concentration of EL studied (21%).

As was shown, HC had a short-term capacity for water retention but almost no capacity for retaining water in the long term.

### 2.7. Comparison of the Studied Properties with Results in Human Skin Reported in the Scientific Literature

Some scientific papers have already stated claims based on efficacy tests that were performed with volunteers for HC and EL ingestion or topical application of cosmetic products containing these two active ingredients. Different clinical trials demonstrated that properties such as hydration, elasticity, transepidermal water loss (TEWL), reductions in wrinkles, and some other aspects of skin aging can be addressed by using these active ingredients. HC was used as both a nutraceutical and a cosmetic ingredient. The oral intake of a hydrolysate of EL showed beneficial effects on human skin, such as improvements in skin elasticity and wrinkles [21,53].

In most of the reported studies of oral ingestion of HC at different dosages, it was shown that improvements in skin elasticity and hydration were more evident than reductions in transepidermal water loss (TEWL) [44,54,55,56,57,58,59,60,61]. However, no effects on increased skin elasticity with the use of topical products with HC were reported. According to Berardesca et al. [62], topical application of HC produced significant improvements in skin hydration, surface smoothness, and luminosity. Ohara et al. [63] reported a dose-dependent effect of oral ingestion of two and a half or ten grams of HC on stratum corneum hydration, but it must be noted that only two dosages were studied. They also highlighted that stratum corneum hydration is the unique beneficial aspect of skin, but they found no significant differences in elasticity or TEWL improvement. In our study, hydration could be related to two properties: pore reduction and moisture retention. A directly proportional relationship was found between pore reduction and the concentrations of HC and EL. Although EL had positive effects on moisture retention in the short term, not enough concentrations were studied to see a clear tendency. In the case of HC, no tendencies were found, but a positive result was obtained for only the highest concentration: 10% HC.

As reported by Maia Campos et al. [64], the combination of topical products and oral supplementation of Col peptides can bring the best benefits for keeping skin in good condition, as their effects can be complementary. Topical products produce a short-term effect, and nutraceuticals have a long-term effect. They also demonstrated the pore miniaturization effect of HC.

## 3. Materials and Methods

### 3.1. Materials

Medium-molecular-weight chitosan extracted from seashell skeletons with a DDA of 85% was acquired from Aldrich, Merck Life Science S.L. (Madrid, Spain) (product number 448877) with a viscosity of 340 cps (200–800 cps) (C = 1%, 1% acetic acid). Acetic acid (glacial) and sodium hydroxide pellets were acquired from PanReac AppliChem, Panreac Química S.L.U. (Barcelona, Spain). Sodium tripolyphosphate with a technical grade of 95.2% was supplied by Alfa Aesar, Thermofisher GmbH (Kandel, Germany). Agarose BioReagent for molecular biology, low electroendosmosis (EEOO), was supplied by Sigma-Merck Life Science S.L. (Madrid, Spain). Phosphate-buffered saline tablets were supplied by Sigma-Merck Life Science S.L. (Madrid, Spain). One tablet of phosphate-buffered saline dissolved in 200 mL of deionized water yielded 0.01 M phosphate buffer, 0.0027 M potassium chloride, and 0.137M sodium chloride with a pH of 7.4 at 25 °C. Calcium chloride from Quimivita, S.A. (Barcelona, Spain) was used in granular form. Teflon molds with a diameter of 6.0 cm were fabricated at the university and were specially designed for the creation of these membranes. The microneedle device was a 140 DRS DermaStamp^®^ system acquired from IBeauyMachine.com GBS International Holding Ltd. (Guangzhou, China). In this work, a Dermastamp^®^ with needles with a depth of 3.0 mm was employed [7]. This depth allowed us to achieve complete perforation of the three layers of the membrane. Customized Franz cells of a special size were designed and fabricated by Fisher Scientific (S.L., part of Thermofisher Scientific (Madrid, Spain)). Bovine elastin from the neck ligament—also in powdered form—was bought from Sigma-Aldrich^®^. Refrigerated storage (2–8 °C) was important for preventing EL degradation. HC is a group of peptides with a low molecular weight (3–6 KDa) [27]. Type I Col is the main Col found in skin, representing 80–90% of skin Col. Type I HC at 5 KDa in powder from fish skin was used. It was bought from Rousselot as the Peptan F5000 HD. According to Rousselot, it presents a random coiled conformation due to its denaturation process. Two commercial vegan “collagen-like” products for cosmetic use with the following compositions (expressed in inci names) were also studied: Veg Col-A: glycerin: 70.0099%, aqua (water): 27.99%, pentylene glycol: 2%, and nicotiana benthamiana hexapeptide-40 sh-polypeptide-47: 0.0001% [65]. Veg Col-B: aqua (water): 93.5%, collagen amino acids: 4.5%, and leuconostoc/radish root ferment filtrate: 2% [66]. Veg Col-A is a type I Col fragment produced in Wild plants as biofactories. According to the supplier’s information on Veg Col-A, a molecule identical to a fragment of the human Col type I sequence could be obtained with the proper post-translational hydroxylations required to be fully functional. Veg Col-B was made from the enzymatic hydrolysis of corn, wheat, and soy proteins.

### 3.2. Methods

#### 3.2.1. Support Preparation

The support for membrane manipulation was based on agarose. The preparation of this support was performed according to a procedure that has been described elsewhere [7]. A 2% solution of agarose was prepared under magnetic stirring and heated up to 80 °C until turning transparent. Then, 9 g of this solution was placed in the Teflon molds for 5 min. Finally, supports are extracted from molds and soaked in a 10% TPP bath for at least 15 min to passivate agarose reactivity, followed by a water bath for another 15 min.

#### 3.2.2. Stock Solution Preparation

Three independent solutions were prepared: acetic acid, NaOH, and TPP were separately dissolved in water, and Ch was added posteriorly to the acetic solution. The following quantities are described for obtaining 116 g of stock solution. The first solution was acetic acid 2% (*v*/*v*) sol. The second solution was 1 g of NaOH dissolved in 25 mL of water. The third solution was a 10% sodium tripolyphosphate (TPP) solution, which needed to be mixed with magnetic stirring until it was solubilized. A total of 3 g of chitosan powder was added to 97 mL of acetic acid solution, followed by the addition of a known quantity of the second solution. Firstly, the solution was stirred manually for about one minute until jellification was obtained. Hereafter, it was stirred automatically with an anaxial flow agitator for 3 h at 542 rpm until homogenization. The final solution pH should be between 5.5 and 5.8 to assure crosslinking. In order to always have 116 g of stock solution weight, a weight correction was made at the end by adding water. It was again stirred with an anaxial flow agitator for 15 min at 542 rpm until homogenization. Finally, a transparent solution was obtained [7].

#### 3.2.3. EL, HC and Veg Col Solution Preparation

The previous stock solution was used to obtain the EL, HC, and Veg Col solutions. Different solutions with different concentrations were used.

EL Solution Preparation

Different solutions of several concentrations were used by adding different concentrations of Bovine elastin from the neck ligament in powder to a known quantity of stock solution. Once the powder was added, automatic stirring was performed for 30 min at 542 rpm for all EL concentrations studied. The resultant solution was homogeneous yet translucid. No increase in the viscosity of the solutions was observed.

HC and Veg Col Solution Preparation

Different solutions were prepared by adding several concentrations of Peptan F5000 HD (identified as HC) in powder to a known quantity of stock solution. Consecutively, automatic stirring was performed for 30 min at 542 rpm for all HC concentrations studied. The obtained solution was transparent for all concentrations of HC. However, an increase in solution viscosity was observed in a concentration-dependent manner for the different solutions. Still, it was observed that the viscosity decreased over time. As an increase in viscosity could create some difficulties during membrane creation, the following procedure was performed: For low concentrations—from 0.1 to 2% HC—the solutions were ready to use from the moment that they were created. From 2.6 to 10% HC, the solutions were left to evolve for three days to two weeks, depending on the concentration, so the viscosity decreased enough to make them easy to pour.

Two vegan “collagen-like” solutions were prepared by adding 2% of each to a known quantity of stock solution. Automatic stirring was performed for 30 min at 542 rpm. A transparent solution was obtained.

EL, HC, and Veg Col solutions at different concentrations were independently tested in different layers of a membrane in order to see their effects on 3L and 2L_(i+b)._

#### 3.2.4. Membrane Preparation

The membrane preparation was conducted at room temperature (21 ± 2 °C).

The preparation of a tri-layered membrane consisted of creating three layers, as previously described [7]. The difference in this work was the addition of the solution containing the active ingredient, or Veg Col product, to 3L or 2L_(i+b)_. Finally, a different curation process was performed to determine whether the base or the activated membrane. The activated membrane differed from the base membrane because some physical pores were created with a microneedle device during the curation process [7].

**Scheme 1:**
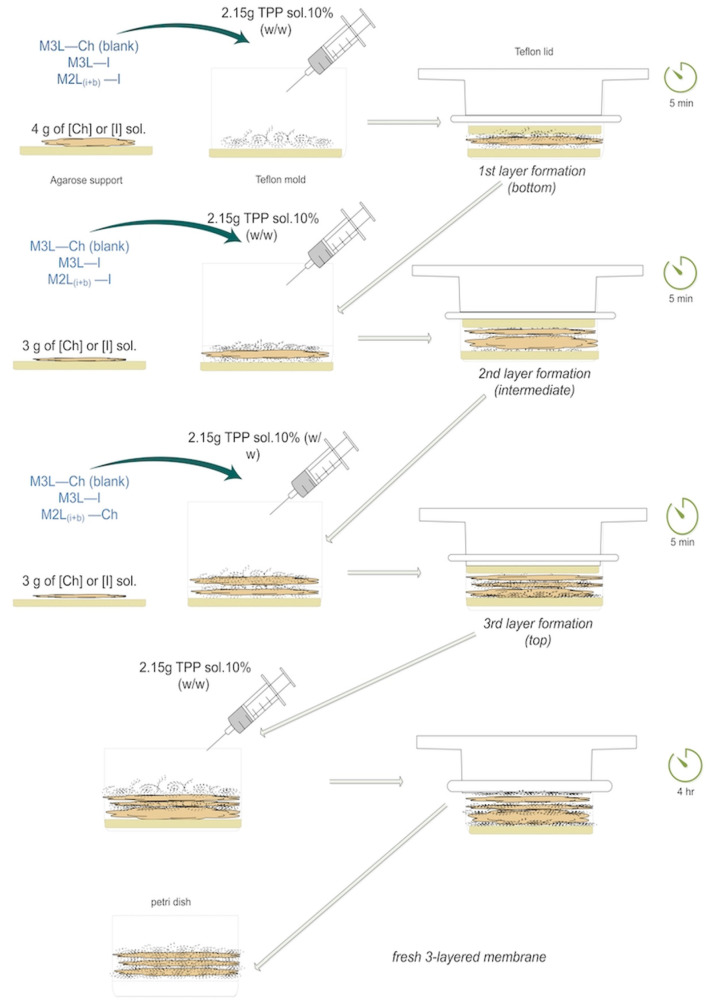
Tri-layered crosslinked membrane formation. The solutions employed by the different layers to obtain the membranes of study are identified in the Scheme as: M3L—Ch (blank) for blank membranes containing the stock solution in the 3L; M3L—I for membranes containing EL, HC, or Veg Col solutions in the 3L; and M2L_(i+b)_—I for membranes containing EL, HC, or Veg Col solutions in the two inner layers. The top layer of M2L_(i+b)_—I was identified by M2L_(i+b)_—Ch, which always contained 2.6% of Ch solution (stock solution).

In Scheme 1, it can be observed the detailed procedure of preparing the different tri-layered membranes with the inclusion of the active ingredients or Veg Col products independently in the three or two inner layers. The first layer, bottom, was prepared by weighting 4 g of Ch or I solution, depending on whether a blank membrane (Ch solution) or a membrane containing EL, HC, or Veg Col (I solution) was performed. The second layer, the inner, was composed of 3 g of Ch (for blank) or I solution. The third layer, the top, was also composed of 3 g of Ch (for blank or M2L_(i+b)_) or I solution. All layers were crosslinked between them with a 10% TPP solution, as detailed elsewhere [7]. Finally, the resulting membrane was left to rest in the mold for 4 h to get consistency, with a Teflon lid on top to obtain flatness. Afterwards, the membrane was weighted and kept in a Petri dish.

It should be stated that the addition of EL, HC, or Veg Col products was made in solution form and always combined with Ch. Hence, these solutions were used as the scaffold for the layer development process when creating the tri-layered membrane and were not added after the tri-layered membrane formation.

**Scheme 2:**
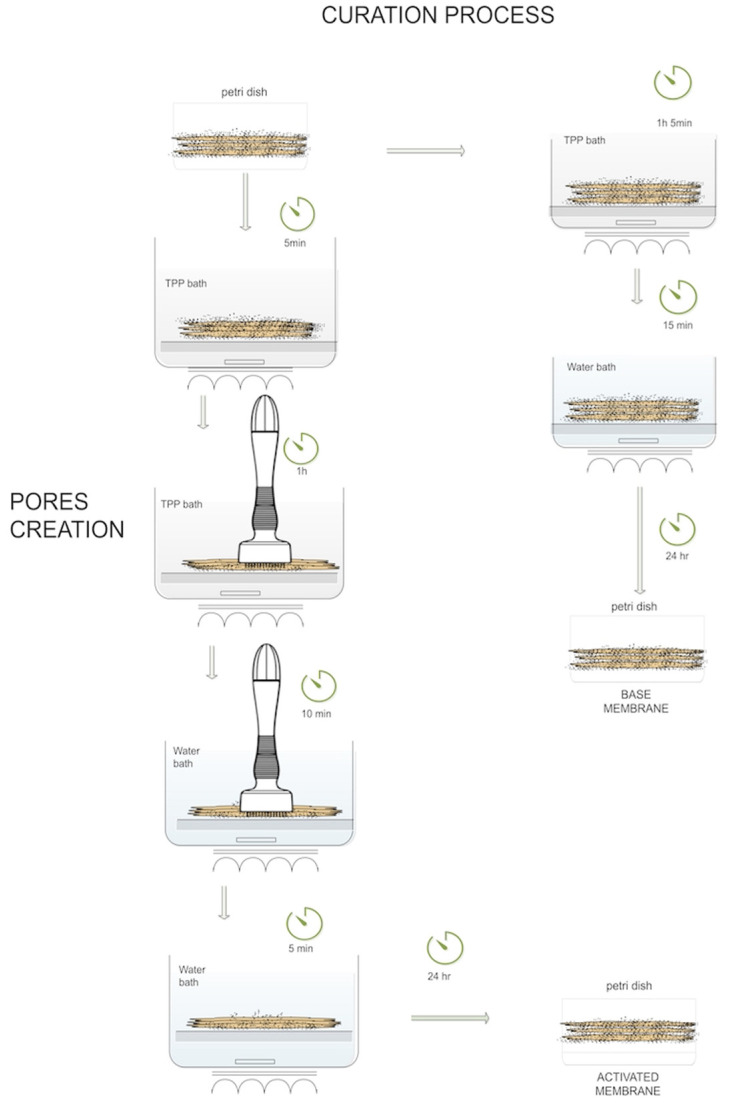
The curing process of activated and base tri-layered membranes is performed for all kinds of membranes [7].

In Scheme 2, it can be observed that the curing process was performed for all membranes obtained after finishing the process described in Scheme 1. For base membranes, they were soaked in a 10% TPP bath for 1 h 5 min followed by a 15 min water bath, in order to stop the TPP coagulation reaction. For activated membrane, after 5 min of TPP bath, the membrane gained some consistency. Following this, the membrane was punched with the microneedles, and it was left in the TPP bath for 1 h. Then, a 10 min water bath was performed with the Dermastamp^®^. Finally, the Dermastamp^®^ was removed, and a 5 min water bath was performed to neutralize the whole activated membrane.

After 24 h, the base, or activated membrane, was ready to use.

Finally, the membranes were cut in order to obtain a round membrane with a diameter of 2 cm.

#### 3.2.5. Characterization of the Different Membranes

The different kinds of membranes were identified as follows: M2L_(i+b)_-I%. First position: M = membrane. Second position: 3L, 2L = number of layers containing the active ingredient or product of study (3 layers or 2 layers); subindex: i = intermediate layer; b = bottom layer if the active ingredient or product was introduced into the two inner layers. Third position I%: active ingredient or product (in this case, HC, EL, or Veg Col product) with the concentration % (*w*/*w*) added to the Ch stock solution. All of the membranes were crosslinked with TPP.

All characterization experiments presented below were carried out with tri-layered crosslinked base membranes, except for the permeation tests and pore quantification tests, where only activated membranes were used.

#### 3.2.6. Concentrations of the Active Ingredients and Veg Col Products

The stock solution contained 2.6% Ch and 0.56% sodium hydroxide. Subsequently, different concentrations of HC or EL were added to this stock solution.

The Ch concentration (%) in the solution when different concentrations of the active ingredient or Veg Col product (I) had been posteriorly added was calculated with Equation (1).

(1) $$\left[Ch\right]\%\;sol=\frac{2.6\;g\;Ch}{\left(100\;g\;sol+I\;g\right)}\times 100$$

where I is the quantity of the active ingredient or product added.

The dry weight of the concentration in the solutions was calculated by considering the following residues in the percentage of the HC or EL solutions: concentration of Ch in %, 0.56% sodium hydroxide, and concentration of the active ingredient in %. The acetic acid and TPP used between the layers had almost negligible amounts of residue. The TPP residue from the bath used during the curation process of the membrane was not calculated, as it could not be determined how much TPP was trapped during the curation process of the membrane. Hence, the TPP absorbed from the bath was not considered.

The concentration of Ch (%) in dry weight, [Ch]% dry weight, when different concentrations of active ingredients were added to the solution was calculated with Equation (2).

(2) $$\left[Ch\right]\%\;dry\;weight=\frac{\left[Ch\right]\%\;sol}{\left(\left[Ch\right]\%\;sol+0.56\%\;NaOH\;sol+\left[I\right]\%\right)\;sol}\times 100$$

The concentration of the active ingredient [I] (%) in dry weight when different concentrations of active ingredients were added to the solution was calculated with Equation (3).

(3) $$\left[I\right]\%\;dry\;weight=\frac{\left[I\right]\%\;sol}{\left(\left[Ch\right]\%\;sol+0.56\%\;NaOH\;sol+\left[I\right]\%\right)sol}\times 100$$

Table 3 and Table 4 show the identification of membranes with the inclusion of I in 3L or 2L_(i+b)_ at the different concentrations studied, with the equivalent of their concentrations in dry weight. When I was included in the 2L_(i+b)_, it was always introduced into the two inner layers, as it is naturally found in skin. The top layer was always a solution of 2.6% Ch (stock solution).

As it was already stated, the concentration of EL in the dry weight of the skin is around 2–4%. This concentration was also studied in this work, with a concentration of 0.085% EL in the solution.

As was previously described, the concentration of Col in the dry weight of the skin is around 75%. This concentration was covered with concentrations of 7.5 and 10% HC.

In order to see if the reduction in Ch concentration when the concentration of HC increased would affect the results, a lowest concentration of Ch sol., 2.1%, was used as a blank to see the effects on elasticity, and no differences in the results were found. For the swelling, permeation, and moisture retention tests, 2.3% Ch blank membranes were obtained to prove that there were no variations in the results in comparison with a 2.6% Ch membrane.

Two commercial products of vegan origin with a “collagen-like” effect were also studied at the concentrations used in the efficacy studies performed by the two different suppliers of the products, as shown in Table 5.

#### 3.2.7. Pore Quantification

The quantification of pores was conducted on activated membranes, as mechanical pore channels were created in these kinds of membranes.

The pores of the tri-layered crosslinked membranes were observed with a Euromex Bioblue Optical Microscope. A semi-plan 4 × 0.10 oil-immersion objective with a wide field of 0.45 was used. The ImageFocus 4 software v. 3.1.2 for Windows, developed by Euromex, from Papenkamp, 6836 BD Arnhem, The Netherlands, was employed to measure the area of the pores. Calibration correction was applied on a computer with the objective that was used. The resolution that was chosen was 2040 × 1528. The pores that were observed were located in the diffusion area of 1.06 cm^2^ of the Franz cell rings, and the number of pores was 52. Periodical pores were optically checked throughout the activated tri-layered crosslinked membrane, as visualized in Figure 1.

Activated membranes employed for permeation tests were first checked with the Optical Microscope.

The mean percentage of pore reduction in comparison with the blank membrane was calculated according to Equation (4).

(4) $$Av\;pore\;\%=\frac{Pore\;area\;\left[I\right]-Pore\;area\;blank}{Pore\;area\;blank}\times 100$$

where Pore area [I] represents the area (µm^2^) of pore size of the activated membrane containing the active ingredient or Veg Col product, and Pore area blank represents the area (µm^2^) of pore size of the blank activated membrane.

#### 3.2.8. Permeation Tests

The permeation tests were conducted at room temperature (21 ± 2 °C).

Customized Franz cells from Fisher Scientific, S.L. (Madrid, Spain) were especially designed for the membranes in order to perform the water permeation tests.

The diffusion area was 1.06 cm^2^. The membrane was fixed between two permeation rings that were 6.0 cm in diameter, and they were collocated between the donor and receptor compartments of the Franz cells [7]. Afterwards, the system was sealed and clamped until tight. Then, 30 g of deionized water was introduced into the donor compartment to determine the permeation over time by collecting the permeated water in the receptor compartment.

The mean percentage of permeation at 1 min in comparison with the blank membrane was calculated according to Equation (5).

(5) $$Av\;\%\;perm=\frac{G\;perm\;[I]-G\;perm\;blank}{G\;perm\;blank}\times 100$$

where G perm [I] represents the grams that permeated through the membrane containing the specific concentration of the active ingredient or Veg Col product, and G perm blank represents the grams of water that permeated through the blank membrane.

#### 3.2.9. Rheological Tests

Rheological tests were only carried out for base membranes with the different layers combined with an AR 2000 ex Rheometer and Rheology Advantage Instrument Control AR, which were both acquired from TA Instruments (New Castle, DE, USA). The conditions were a frequency of 1 Hz, a strain between 0.01 and 100%, a 20 mm steel cross-hatched (CH) plate, and a temperature of 25 °C. In the test, the membrane was placed on top of the lower geometry, and the rheometer’s motor decreased the upper geometry until the normal force applied was around 1.5 N. The elastic modulus of the material was obtained with an oscillatory procedure.

All elasticity results for the membranes with different concentrations of active ingredients or Veg Col products obtained at different temperatures were normalized with respect to an elasticity result obtained from an interpolated blank membrane in Equation (6), which was obtained from Figure 9, according to the same room temperature of the membrane under study:

(6) $$G^{\prime }\;blank\left(Pa\right)=4525\times T\left({}^{\circ}C\right)-41,022$$

The percentage difference in the elasticity of the active ingredient or Veg Col membrane at the different concentrations [I] with respect to the blank interpolated in Equation (6) was obtained with Equation (7):

(7) $$Elasticity\left[I\right]\left(\%\right)=\frac{Av\;G^{\prime }\left[I\right]\;(Pa)-Av\;G^{\prime }\;blank\;(Pa)}{Av\;G^{\prime }\;blank\;(Pa)}$$

#### 3.2.10. Swelling Index

After the creation of the membranes, the samples were dried with granular calcium chloride for 24 h in a container. In our previous research, we used 24 h after the creation of the membranes until they were dried with calcium chloride [7]. In this work, it was seen that this time could be avoided, as it did not affect the results. After 24 h in the dryer, the membrane weight was constant, and then it was assured that the samples were completely dried.

The swelling tests were conducted at room temperature (21 ± 2 °C).

The dried membranes containing the different concentrations of active ingredient or Veg Col product were weighed and then immersed in phosphate-buffered solution (PBS, pH 7.4) for different lengths of time (0, 30 min, 1 h, 2 h, 12 h, and 24 h) until they reached the maximum weight [7]. The phosphate-buffered solution was prepared as previously reported. The phosphate-buffered solution was under constant agitation during the entire experiment.

An electronic balance was used to weigh the swollen membranes after the surface water was removed with filter paper. Then, the swelling index (SI) of the membranes was calculated according to Equation (8):

(8) $$SI\left(\%\right)=\frac{Ws-Wd}{Wd}\times 100$$

where Ws and Wd were the weights, in grams, of the swollen and dried membranes, respectively.

The swollen weight value was obtained as the maximum weight obtained during the different periods of measurement—up to 24 h (Ws).

The percentage difference in swelling of the active ingredient or Veg Col membrane [I] with respect to the blank membrane was obtained with Equation (9):

(9) $$SI\;\left[I\right]\left(\%\right)=\frac{SI\;\left[I\right]-SI\;\left(blank\right)}{SI\;(blank)}\times 100$$

#### 3.2.11. Moisture Retention

A DBS moisture analyzer from Kern was used to determine the retention of humidity in each membrane. The following program was used: A ramp in the temperature was applied for the first 5 min until 100 °C was reached. The defined temperature was the lamp temperature. The weight loss of the membrane was evaluated every minute for 30 min. The result could be directly related to the capacity of the membrane to retain water. In human skin, this is known as the barrier effect.

In this test, the moisture retention of the whole membrane was evaluated, not just the water retention from the surface (barrier effect), as the membrane was wrinkled when it dried. Therefore, the bottom, which simulated the inner layer of skin and was not exposed to air, was also exposed to the same environmental dryness. Hence, due to the conditions of this test, only the membranes containing the ingredient in 3L were tested.

The moisture retention (MR) (%) was normalized versus the blank membrane according to Equation (10).

(10) $$MR\left(\%\right)=\frac{\left(Av\;blank\;{WL\%}_{t}-Av\;\left[I\right]\;{WL\%}_{t}\right)}{Av\;blank\;{WL\%}_{t}}\times 100$$

where Av blank WL and Av [I] WL were the water-loss values from the blank membrane and from the membrane containing the specific concentration of the active ingredient, or Veg Col product, respectively. The subindex t was the time of measurement—15 or 30 min—of the water-loss percentage of the membrane.

## 4. Conclusions

In this work, some properties of tri-layered membranes containing different active ingredients—specifically, elastin, hydrolyzed collagen, and two different commercial vegan alternatives to collagen—were studied. Based on the results of this study, it can be stated that these membranes were sensitive to the studied properties: pore and water permeation reduction, elasticity, swelling, and moisture retention.

1. These properties were sensitive to the concentration of the active ingredient and the vegan collagen-like product. For the different properties that were studied, different results were obtained. Hence, the effects of the active ingredients and the vegan collagen-like product could be quantified.
2. It was demonstrated that the effects of some active ingredients, namely EL, HC, and two Veg Col products, were shown in the form of pore reduction, water permeation reduction, elasticity, swelling, and moisture retention. Hence, anti-aging claims could be proven.
3. The effects of the active ingredients and vegan collagen-like products on the previous properties varied depending on the layers into which they were introduced. Depending on whether EL, HC, or Veg Col was introduced into the three layers or into the target layers, in our case, the two inner layers, boosted or reduced efficacy was observed for the properties that were studied.

The main results obtained for the different measured properties were:

For permeation tests and its relationship with Pore Reduction:

- The permeation reduction was found to be directly proportional to the concentrations of EL and HC.
- Pore reductions were measured for some concentrations of EL and HC, and a correlation with permeation reduction was found. For similar values of reductions in pores between the two active ingredients, a more significant permeation reduction was observed for EL because of its hydrophobic segments.

For Elasticity tests:

- In general, an inversely proportional relationship between concentration and elasticity was found for both EL and HC.
- The greatest booster effect in the elasticity was found when including a 0.085% of EL in 2L_(i+b)_ (71%). This 0.085% concentration of EL fell within the margins of EL weight in human dermal skin.
- A boost in elasticity was found for the lowest concentration of HC studied (0.1%), and this was greater when HC was included in 2L_(i+b)_ (40%).

For Swelling tests:

- In the case of EL, at extreme concentrations, a boost in swelling was obtained in 3L with respect to 2L_(i+b)_.
- In the case of HC, at extreme concentrations, a boost in swelling was obtained in 2L_(i+b)_ with respect to 3L.

For Moisture retention tests:

- For the two concentrations of EL that were studied, some noticeable moisture retention was observed, and the highest value was observed at the higher concentration (0.27%) at 15 min (short term) (21%).
- For the four concentrations of HC that were studied, an increase in moisture retention was obtained only for the highest concentration studied (10% HC) at 15 min (short term) (19%).

## Figures and Tables

**Table 1:** Mean pore areas (µm^2^) with the standard deviation (SD) and mean percentage of pore reduction (%) in blank activated membranes and in activated membranes containing different concentrations of EL and HC and in different layers vs. blank.

ID M	Nº Pores	Area	SD	Pore Reduction
M3L—Ch (blank)	35	39,045	2975	—
M3L—0.085EL	35	37,120	3910	−4.9
M3L—0.28EL	35	32,204	4452	−18
M2L_(i+b)_—0.085EL ^1^	35	38,856	3743	−0.48
M2L_(i+b)_—0.28EL ^1^	35	31,978	2678	−18
M3L—2.6HC	35	33,091	4467	−15
M3L—10HC	35	30,508	4867	−22
M2L_(i+b)_—2.6HC ^1^	35	38,216	4122	−2.1
M2L_(i+b)_—10HC ^1^	35	35,722	4032	−8.5

^1^ The top layer of membranes containing the active ingredient in the two inner layers (M2L_(i+b)_) was composed of only Ch 2.6% (stock solution).

**Table 2:** Mean permeated grams of water at 1 min with SD and relationship between the mean percentage of water permeation reduction (%) and the mean percentage of pore size reduction (%) for EL and HC vs. blank at 1 min.

ID M	Water Permeation ± SD	Water Permeation Reduction	Pore Reduction
M3L—Ch (blank)	15 ± 2.6	—	—
M3L—0.085EL	12 ± 2.8	−23	−4.9
M3L—0.28EL	5.3 ± 0.07	−65	−18
M2L_(i+b)_—0.085EL ^1^	14 ± 1.7	−10	−0.48
M2L_(i+b)_—0.28EL ^1^	6.1 ± 1.1	−60	−18
M3L—2.6HC	11 ± 3.1	−25	−15
M3L—10HC	9.3 ± 1.7	−39	−22
M2L_(i+b)_—2.6HC ^1^	14 ± 5.5	−9.3	−2.1
M2L_(i+b)_—10HC ^1^	12 ± 1.4	−21	−8.5

^1^ The top layer of membranes containing the active ingredient in the two inner layers (M2L_(i+b)_) was composed of only Ch 2.6% (stock solution).

**Table 3:** Identification (ID) of different membranes with the percentage of EL inclusion in 3L or 2L_(i+b)_ and the concentrations (%) (*w*/*w*) of Ch and EL in solution and in dry weight.

ID M3L	ID M2L	Ch %	EL %	Ch % (Dry w)	EL % (Dry w)
M3L—Ch (blank)	—	2.6	—	82	—
M3L—0.085EL	M2L_(i+b)_—0.085EL ^1^	2.6	0.085	80	2.6
M3L—0.23EL	M2L_(i+b)_—0.23EL ^1^	2.6	0.23	77	6.7
M3L—0.27EL	M2L_(i+b)_—0.27EL ^1^	2.6	0.27	76	7.8
M3L—0.28EL	M2L_(i+b)_—0.28EL ^1^	2.6	0.28	75	8.3

^1^ The top layer of membranes containing the active ingredient in the two inner layers (M2L_(i+b)_) was composed of only Ch 2.6% (stock solution).

**Table 4:** Identification (ID) of different membranes with the percentage of HC inclusion in 3L or 2L_(i+b)_ and the concentrations (%) (*w*/*w*) of Ch and HC studied in solution and in dry weight.

ID M3L	ID M2L	Ch %	HC %	Ch % (Dry w)	HC % (Dry w)
M3L—Ch (blank)	—	2.6	—	82	—
M3L—0.1HC	M2L_(i+b)_—0.1HC ^1^	2.6	0.1	80	3.1
M3L—0.2HC	M2L_(i+b)_—0.2HC ^1^	2.6	0.2	77	6.0
M3L—0.4HC	M2L_(i+b)_—0.4HC ^1^	2.6	0.4	73	11
M3L—2HC	M2L_(i+b)_—2HC ^1^	2.5	2	50	39
M3L—2.6HC	M2L_(i+b)_—2.6HC ^1^	2.5	2.6	44	46
M3L—4HC	M2L_(i+b)_—4HC ^1^	2.5	4	35	57
M3L—7.5HC	M2L_(i+b)_—7.5HC ^1^	2.4	7.5	23	72
M3L—10HC	M2L_(i+b)_—10HC ^1^	2.3	10	18	78

^1^ The top layer of membranes containing the active ingredient in the two inner layers (M2L_(i+b)_) was composed of only Ch 2.6% (stock solution).

**Table 5:** Identification (ID) of different membranes with the percentage of inclusion of Veg Col types A and B in 3L or 2L_(i+b)_ and the concentrations (%) (*w*/*w*) of Ch, Veg Col-A, and Veg Col-B in solution.

ID M3L	ID M2L	Ch %	ID I	I %
M3L—Ch (blank)	-	2.6	-	-
M3L—2Veg Col-A	M2L_(i+b)_—2Veg Col-A ^1^	2.5	Veg Col-A	2
M3L—2Veg Col-B	M2L_(i+b)_—2Veg Col-B ^1^	2.5	Veg Col-B	2

^1^ The top layer of membranes containing the Veg Col product in the two inner layers (M2L_(i+b)_) was composed of only Ch 2.6% (stock solution).

## Data Availability

No new data were created or analyzed in this study. Data sharing is not applicable to this article.
